# Teaching percutaneous renal biopsy using unfixed human cadavers

**DOI:** 10.1186/s12882-015-0210-6

**Published:** 2015-12-11

**Authors:** Scott W. Oliver, Rajan K. Patel, Khalid A. Ali, Colin C. Geddes, Bruce MacKinnon

**Affiliations:** School of Medicine, University of Glasgow, Scotland, UK; Renal and Transplant Unit, Queen Elizabeth University Hospital, Glasgow, Scotland UK; Medical Education, NHS Lanarkshire, Bothwell, Scotland UK; Radiology Department, Queen Elizabeth University Hospital, Glasgow, Scotland UK; Clinical Skills Anatomy Centre, University of Glasgow, Scotland, UK; Postgraduate Office, Wishaw General Hospital, 50 Netherton Street, Wishaw, ML2 0DP UK

**Keywords:** Kidney biopsy, Nephrology, Haemorrhage, Cadaveric simulation

## Abstract

**Background:**

Percutaneous renal biopsy (PRB) is an important diagnostic procedure. Despite advances in its safety profile there remains a small but significant risk of bleeding complications. Traditionally, operators train to perform PRB through tutor instruction and directly supervised PRB attempts on real patients. We describe an approach to teaching operators to perform PRB using cadaveric simulation.

**Methods:**

We devised a full day course hosted in the Clinical Anatomy Skills Centre, with places for nine candidates. Course faculty consisted of two Consultant Nephrologists, two Nephrology trainees experienced in PRB, and one Radiologist.

Classroom instruction included discussion of PRB indications, risk minimisation, and management of complications. Two faculty members acted as models for the demonstration of kidney localisation using real-time ultrasound scanning. PRB was demonstrated using a cadaveric model, and candidates then practised PRB using each cadaver model.

**Results:**

Written candidate feedback was universally positive. Faculty considered the cadaveric model a realistic representation of live patients, while the use of multiple cadavers introduced anatomical variation.

**Conclusions:**

Our model facilitates safe simulation of a high risk procedure. This might reduce serious harm associated with PRB and improve patient safety, benefiting trainee operators and patients alike.

## Background

Percutaneous renal biopsy (PRB) is an essential diagnostic test in renal medicine. Despite advances in safety following the adoption of spring-loaded devices and real-time ultrasound guidance, there remains a small but significant risk of bleeding following PRB [1, 2]. In our unit in 2014 we performed 186 native renal biopsies, equivalent to 129 per million population per year [3]. Our major complication rate is 1.9 % [1], defined as patient death or bleeding requiring blood transfusion, radiological or surgical intervention. The rate of major bleeding complications in the literature varies between 0.4 [4] and 8 % [5].

Competence in performing PRB of native and transplanted kidneys is a requirement of Nephrology Fellowship programmes in the United States [6] and an optional component for Renal Medicine trainees in the United Kingdom [7].

Traditionally, operators are taught to perform PRB through a combination of tutor instruction, clinical observation and supervised PRB attempts in real patients. This approach provides clinical authenticity but presents several disadvantages. Novice or incompetent operators may increase the risk profile and may fail to obtain an adequate biopsy sample. Patients find this unacceptable [8]. Furthermore, experiential learning requires the trainee to obtain relatively frequent practical experience [9], which can be difficult where multiple trainees are competing for experience within a single centre.

Some authors have developed simulation models to teach PRB, including the use of embalmed cadavers [10, 11], although to our knowledge this approach has not been widely adopted. Optimal simulation requires a high degree of functional correlation with the simulated clinical scenario and effective transfer of learned skills into subsequent clinical practice [12]. We describe a novel model for teaching PRB using unfixed human cadavers.

## Methods

We devised a full-day course hosted at the Clinical Anatomy Skills Centre, a state-of-the-art anatomy laboratory operated jointly by the University of Glasgow and the Royal College of Physicians and Surgeons of Glasgow. Course faculty consisted of two consultant nephrologists, two nephrology trainees experienced in PRB, and one radiologist. On the third run of the course a consultant pathologist joined the faculty to demonstrate correct handling of a renal biopsy tissue core and to confirm, using a dissecting microscope, that tissue obtained by candidates was indeed renal cortex. The structure of the day was designed to focus upon the acquisition of practical skills, augmented with classroom discussions of the indications for PRB and the management of potential complications. The course format is presented in Table 1.

**Table 1:** Timetable for the course

09:30	Registration and introductions
10:00	Biopsy indications
10:15	Ultrasound localisation of the kidney
11:15	Coffee
11:30	Ultrasound guided biopsy demonstration (cadavers)
13:00	Lunch
13:30	Biopsy complications
14:00	Simulated real-time biopsy (cadavers)
16:00	Closing remarks

**Fig. 1:**
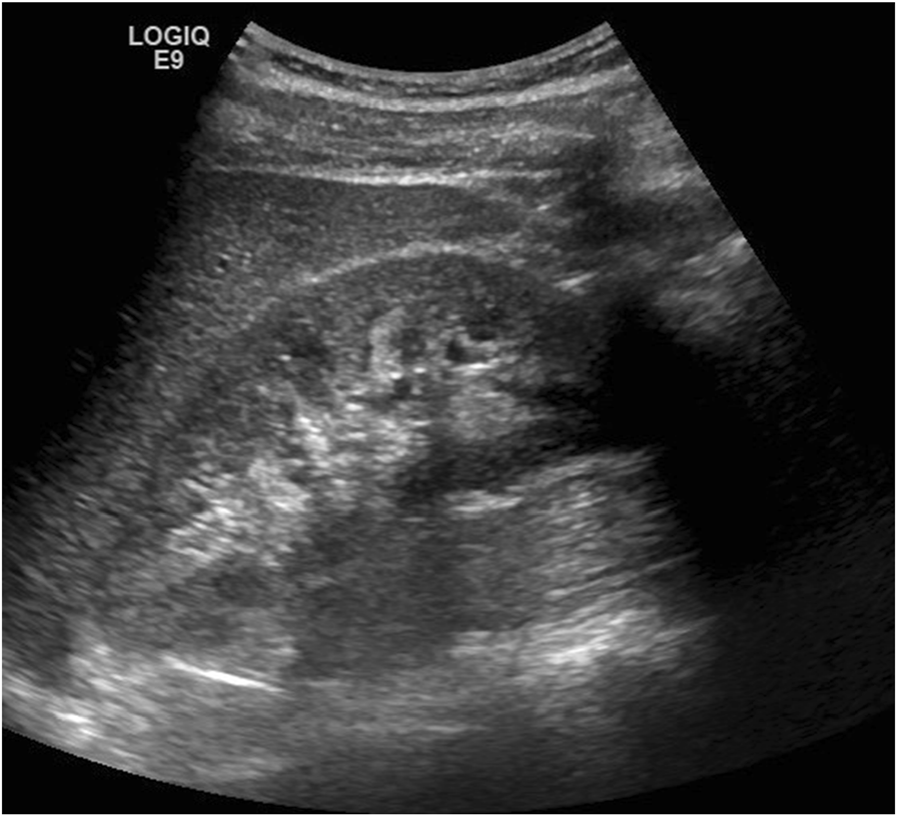
Ultrasound localisation of kidney in live model

**Fig. 2:**
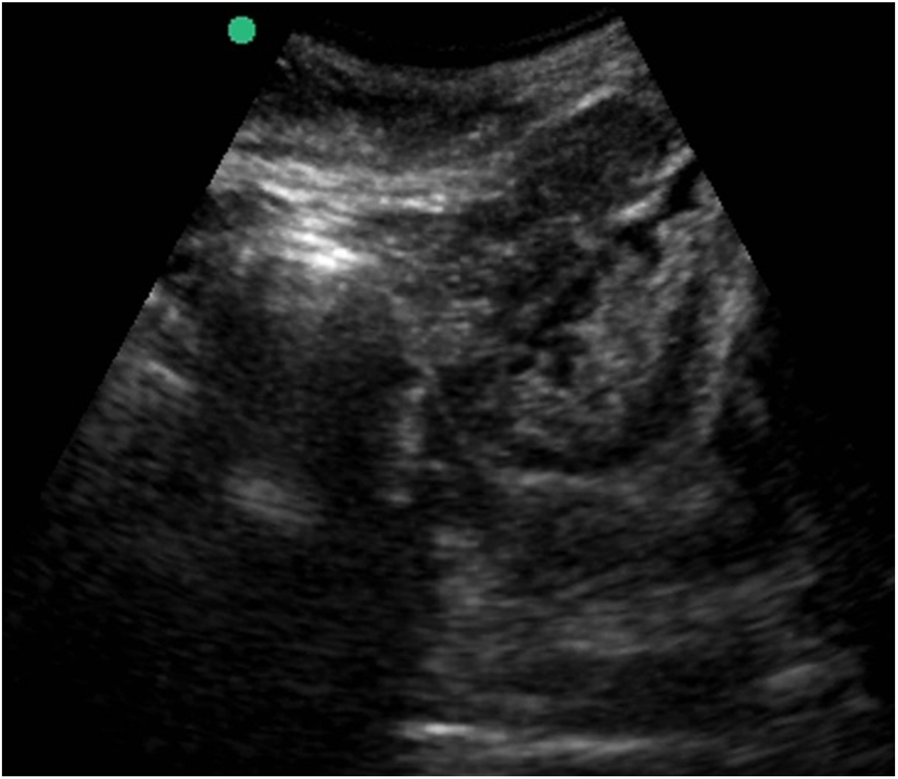
Ultrasound localisation of cadaveric kidney

Following initial classroom discussion we proceeded to demonstrate the ultrasound localisation of native kidneys in life (Fig. 1), using faculty members as models. Candidates practised this technique under faculty supervision, then compared the ultrasound images with those of cadaveric models (Fig. 2). We subsequently demonstrated PRB using Peyton’s four-step approach, a validated means of teaching technical skills [13]. Candidates then had opportunities to practise PRB using each of three cadaveric models under faculty supervision.

Written candidate feedback was gathered at the conclusion of the course on an anonymous, voluntary basis.

The Clinical Anatomy Skills Centre Management Group approved the course outline and the use of cadaveric material. The University of Glasgow supplied the cadaveric material. This was obtained through donations to the University of Glasgow Anatomical Bequest Programme, which operates under the terms of the Anatomy Act. Approval was given by the Lead Licence Holder of the University of Glasgow to submit a description of this educational course for publication, including the use of cadaveric images captured on ultrasound scanning.

## Results

Unfixed human cadavers provide a useful model for teaching PRB. Cadaveric kidneys have near-realistic ultrasound appearances (Fig. 2) with realistic anatomy and tissue consistency. Biopsy specimens obtained by candidates had a realistic macroscopic appearance reflecting successful and unsuccessful attempts at PRB. The use of multiple cadavers also introduced natural anatomical variation, and the ability for repeated practise on left and right native kidneys.

Our course has run three times with six, ten and eight candidates respectively. There is capacity to accommodate up to ten candidates using five cadavers. Our candidates were mostly nephrology trainees, but also included consultant nephrologists and non-nephrology medical trainees. Candidate evaluation data were universally favourable, with every candidate stating they were either “pleased” or “very pleased” with every individual aspect of the course. Free text comments indicated particular enthusiasm for the clinical authenticity provided by the cadaveric model (Table 2).

**Table 2:** Candidates’ feedback on the course highlights

“Being able to practice renal biopsy from start to finish in in real time. Adequate time for practical session for each person. Use of cadavers to simulate real life as much as possible.” “Hands on experience. Getting used to the feel of biopsies.” "Student to tutor/cadaver ratio was brilliant. Ample time to practise on cadavers" "I gained experience and feel more confident" "This was an amazing hands-on experience. Brilliant teaching. I also loved the demonstration video that was made available before the course" “USS localisation of kidney and hands on experience of renal biopsy.” “Very useful to have so much practice and try all cadavers for variations.” “Opportunity to have a go at multiple biopsies. Equivalent of several weeks’ opportunity in practice.” “Excellent ultrasound image. Useful to try on 8 kidneys.”	

## Discussion

There are several advantages to using this model to teach PRB. Tuition is delivered without any risk to patient safety, and multiple operators can be trained in the same session using the same faculty resources.

The cadaveric model provides an authentic and realistic means of teaching PRB, and is in keeping with best practice in clinical simulation teaching [12]. The model is more realistic than other described simulators, and through using multiple cadavers the candidate can acquire genuine technical skills instead of just mastering the simulator. Table 3 compares this model with other reported forms of simulated PRB.

**Table 3:** Comparison of simulated models for teaching PRB

Technique	Advantages	Disadvantages
Unfixed human cadavers (cost £3500 GBP/$5250 USD for 10 participants including tuition, catering, associated technical costs of providing course)	Clinical authenticity; Life-like ultrasound imaging; Authentic biopsy specimen; Normal anatomical variation	Relatively high cost; Specialist facilities needed to store cadaveric material
Porcine kidney within turkey breast [10] (cost £13 GBP/$20 USD)	Low cost; Portable to peripheral sites; Near-authentic biopsy specimen	Technically challenging to create model; Limited clinical authenticity
Cryopreserved porcine kidneys [15] (cost unknown)	Low cost; Portable to peripheral sites Near-authentic biopsy specimen	Limited clinical authenticity; Requires euthanised animals; Perfusion fluid leaks can limit model usability
Synthetic torso with synthetic kidney [16] (cost £1300 GBP/$2000 USD per torso; £40-250 GBP/$60-375 USD per kidney)	Clinical authenticity; Portable to peripheral sites	Relatively high cost; Ongoing requirement for synthetic materials from commercial suppliers.

Potential disadvantages of the cadaveric model include the lack of kidney movement with respiration, and the inability to determine the adequacy of analgesia. These were not considered major flaws by faculty or course attendees.

Unfixed cadaveric material is relatively expensive to use for this purpose. Unfixed cadavers are not fixed with chemical preservatives, but instead are frozen at the time of donation and thawed just prior to use. This provides significantly more realistic tissue consistency than fixed cadaveric material. When the cycle of freezing and thawing the unfixed cadaver has been repeated three or four times, the material becomes unsuitable for further simulator use. In contrast, fixed cadaveric material provides a less realistic simulation model, but the tissue remains in a suitable condition for use for several months or longer.

The course costs must be viewed in the context of the potential financial, medical and ethical costs of current practice for teaching PRB with an ad hoc experiential approach. As well as reduced educational efficacy, “see one, do one” carries significant potential to cause patient harm, or result in an inadequate biopsy specimen being obtained.

Candidates valued the opportunity to gain significant clinical experience with PRB during the course. Such training is known to increase candidate confidence in performing PRB and may also reduce major complications [14]. Given our low baseline major complication rate [1] it is challenging to assess the clinical impact of this course. We continue to monitor biopsy adequacy and complication rates through the Scottish Renal Biopsy Registry (part of the broader Scottish Renal Registry) [3].

## Conclusions

In conclusion, unfixed human cadavers provide an excellent simulation model for teaching PRB in a way that is acceptable to patients and clinicians. We plan to run this course on a regular basis, and encourage other renal units to adopt similar approaches to optimising clinical training for high risk procedures.

## Abbreviations

PRB: percutaneous renal biopsy
